# Can palliative radiotherapy influence prostate-specific antigen response in patients with castrate-resistant prostate cancer treated with systemic therapy (chemotherapy or abiraterone)?—a report of three cases

**DOI:** 10.7497/j.issn.2095-3941.2014.0025

**Published:** 2015-03

**Authors:** Mohan Hingorani, Sanjay Dixit, Pattu Pugazhenthi, Simon Hawkyard, Andrew Robertson, Richard Khafagy

**Affiliations:** 1 Department of Clinical Oncology, Castle Hill Hospital, Hull and East Yorkshire, NHS Trust, Cottingham, East Riding of Yorkshire, HU16 5JQ, UK; 2 Department of Urology, Scarborough District General Hospital, Scarborough ON M1P 2V5, UK

**Keywords:** Prostate cancer, palliative, radiotherapy, chemotherapy

## Abstract

Palliative radiotherapy (pRT) is primarily employed for palliation of bone pain in patients with castrate-resistant prostate cancer (CRPC). However, evidence that pRT influences prostate-specific antigen response in patients with CRPC on systemic therapy is lacking. We describe three cases of CRPC progressing after treatment with docetaxel (*n*=2) and abiraterone (*n*=1), who responded unusually after pRT for bone pain with the development of a significant biochemical response and restoration of response to systemic therapy. The possibility of pRT influencing metastatic disease in CRPC has not been previously reported, and raises the possibility of radiation-induced modulation of anti-tumor immune response mechanisms that may play a role in the restoration of response to systemic treatment.

## Introduction

The last decade has witnessed the development of several novel therapeutic strategies for the management of castrate-resistant prostate cancer (CRPC); these strategies include abiraterone acetate, enzalutamide, immunomodulatory therapy (sipuleucel-T), cabazitaxel, and radium-223^1–3^. However, the role of palliative radiotherapy (pRT) in castrate-resistant disease remains poorly defined. pRT is often employed for the palliation of metastatic bone lesions in CRPC, resulting in a relief of pain in about 80%-90% of patients combined with a reduction in analgesic requirement^4^. However, published evidence on the induction of a prostate-specific antigen (PSA) response following pRT for metastatic bone disease is lacking. In this report, we describe three cases of CRPC developing biochemical progression upon systemic chemotherapy and abiraterone in whom fractionated pRT to bone lesions was followed by induction of PSA response and restoration of response to systemic treatment.

## Case reports

All patients had castrate-resistant disease which previously progressed on luteinizing hormone-releasing hormone (LHRH) analogue therapy and maximal androgen blockade (MAB), and two patients had also previously received diethylstilbestrol. All patients had received at least two previous lines of therapy. Two patients were receiving salvage treatment with palliative chemotherapy using docetaxel and one patient was on abiraterone acetate. All patients were also receiving synchronous prednisolone, bisphosphonate (zoledronic acid), and LHRH analogue therapy. Patient and disease-related characteristics combined with initial management and subsequent lines of therapy are summarized in **Table 1**.

**Table 1:** Patient and disease-related characteristics combined with initial management and subsequent lines of therapy

Item	Case 1	Case 2	Case 3
Diagnosis and management (first-line therapy)	• 64 years	• 72 years	• 78 years
	• August 2010	• March 2008	• November 2010
	• PSA =450 ng/mL	• PSA =1,170 ng/mL	• PSA =1,400 ng/mL
	• Skeletal metastasis	• Skeletal metastasis	• Skeletal metastasis
	• Bicalutamide monotherapy	• LHRH analogue therapy	• LHRH analogue + bicalutamide (MAB)
	• Zoledronic acid infusions	• PSA nadir 4.3 in September 2009	• PSA nadir 36.5 in November 2011
	• PSA nadir in November 2010		
	• Palliative radiotherapy (spine)		
Second-line therapy	• November 2011	• March 2010	• May 2012
	• PSA =23 ng/mL	• PSA =16.3 ng/mL	• PSA =1,400 ng/mL
	• LHRH analogue therapy	• LHRH analogue + bicalutamide (MAB)	• LHRH analogue + DES
Third-line therapy	• September 2012	• September 2010	• October 2013
	• PSA =77 ng/mL	• PSA =46.5 ng/mL	• PSA =868 ng/mL
	• LHRH analogue + bicalutamide (MAB)	• LHRH analogue + DES	• Pelvic nodes + skeletal metastasis
	• Zoledronic acid infusion	• Zoledronic acid infusion	• LHRH analogue + docetaxel
			• Prednisolone
			• Zoledronic acid infusion
Fourth-line therapy	• May 2013	• April 2012	
	• PSA =81 ng/mL	• PSA =273 ng/mL	
	• No visceral metastasis	• No visceral metastasis	
	• LHRH analogue + docetaxel	• LHRH analogue + docetaxel	
	• Prednisolone	• Prednisolone	
	• Zoledronic acid infusion	• Zoledronic acid infusion	
Fifth-line therapy		• June 2013	
		• PSA =628 ng/mL	
		• No visceral metastasis	
		• LHRH analogue + abiraterone	
		• Prednisolone	
		• Zoledronic acid infusion	

PSA, prostate specific antigen; LHRH, luteinizing hormone-releasing hormone; MAB, maximal androgen blockade; DES, diethylstilboestrol.

**Figure 1:**
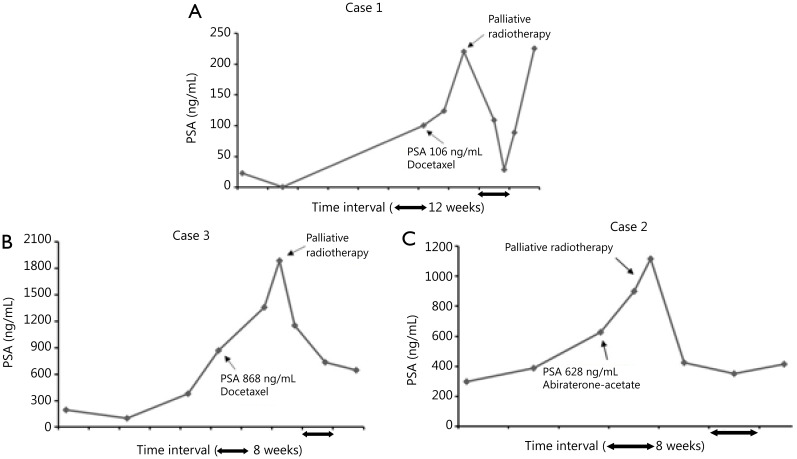
PSA kinetics before and after fractionated pRT that was delivered during systemic treatment with docetaxel chemotherapy (A and B) and abiraterone (C). PSA, prostate specific antigen; pRT, palliative radiotherapy.

Patients receiving docetaxel chemotherapy received up to four cycles (three weekly), and the patient on abiraterone acetate received 10 weeks of treatment. All patients showed evidence of symptomatic and rapid biochemical progression with increasing PSA velocity and doubling time of less than 6 weeks, thereby raising a strong argument for the discontinuation of salvage systemic treatment. However, all patients were referred for fractionated pRT (20 Gy in 5 fractions) because of increasing bone pain. Two patients received radiotherapy to the spine, and one patient received radiotherapy to the pelvis. Chemotherapy was interrupted during radiotherapy, and docetaxel was recommenced in patients at least 2 weeks after completion of radiotherapy. However, the patient receiving abiraterone acetate continued with treatment during radiotherapy. The PSA kinetics during systemic treatment and following completion of radiotherapy are displayed in **Figure 1**.

**Figure 2:**
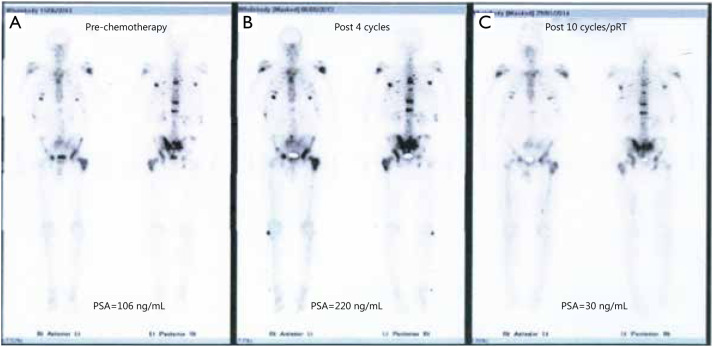
Bone scan appearances (case 1) during the treatment course demonstrate response to systemic chemotherapy after pRT for metastatic bone disease. (A) Bone scan prior to starting chemotherapy (PSA =106 ng/mL); (B) Bone scan after four cycles of chemotherapy showed no change, but patient developed increasing bone pain with rapid biochemical progression (PSA =220 ng/mL); (C) Bone scan performed after pRT, and further six cycles of chemotherapy showed a significant reduction in uptake (PSA =30 ng/mL). pRT, palliative radiotherapy; PSA, prostate specific antigen.

Following completion of radiotherapy, a dramatic biochemical response was observed with a marked reduction in PSA levels in all patients, which was associated with symptomatic response and clinical improvement. The biochemical response after radiotherapy was striking, with PSA reducing to 30%-60% from baseline peak levels within 6 weeks of completion of treatment. All patients recommenced previous systemic therapy, and subsequent treatment cycles were associated with preserved biochemical response and disease stabilization on repeat staging investigations (**Figure 2**). In addition, all patients experienced symptomatic benefits with resolution of bone pain and improvement in performance status and overall quality of life. Two patients remained in biochemical remission 4 and 7 months after completion of pRT, and the remaining patient developed biochemical relapse at 8 months after completion of radiotherapy. The two patients receiving docetaxel completed the originally intended 10 cycles of chemotherapy, and the remaining patient remained on abiraterone more than 12 months after initiation of therapy.

## Discussion

The possibility of pRT influencing metastatic disease has been reported previously in the context of metastatic melanoma^5^. Radiotherapy has been shown to have immunomodulatory effects, with radiation-induced cell death inducing the release of endogenous danger signals known as damage-associated molecular patterns, which may augment the presentation of tumor antigens released from necrotic tumor cells in the presence of inflammatory milieu characterized by neutrophils, lymphocytes, natural killer cells, and increased systemic levels of cytokines (i.e., interleukin-6 and tumor necrosis factor). Therefore, radiation can promote the development of tumor-specific immune-active phenotype with initiation of an “abscopal” bystander effect at distant sites from radiation^6^. Ludgate *et al*.^7^ hypothesized that these mechanisms may be accentuated in prostate cancer in the presence of an androgen-deprived environment. Radiation-mediated immunological upregulation in prostate cancer tumor cells may be enhanced in the presence of fractionated radiotherapy compared with single fraction^8^.

The aforementioned cases demonstrated remarkable similarity in terms of advanced castrate-resistant disease and signs of rapid disease progression on systemic therapy prior to fractionated pRT for metastatic bone disease. Subsequently, these patients demonstrated an excellent response with marked reduction in PSA levels that matched the rate of progression and PSA velocity prior to radiotherapy. The probability of delayed PSA response to systemic therapies remains a distinct possibility in this situation. However, the paradoxical and extremely rapid levels of PSA reduction are relatively uncommon in the context of systemic treatment of prostate cancer. The timing and unusual nature of the PSA response does raise the provocative probability of likely association with immune stimulatory effects of pRT that may promote a synergistic interaction between radiation and systemic therapy. Indeed, the possible existence of such a phenomenon may enable the exploration of several novel therapeutic strategies in the future management of CRPC.
